# Organoids and microphysiological systems: Promising models for accelerating AAV gene therapy studies

**DOI:** 10.3389/fimmu.2022.1011143

**Published:** 2022-09-26

**Authors:** Ritu Mahesh Ramamurthy, Anthony Atala, Christopher D. Porada, Graҫa Almeida-Porada

**Affiliations:** Fetal Research and Therapy Program, Wake Forest Institute for Regenerative Medicine, Winston-Salem, NC, United States

**Keywords:** AAV, gene therapy, AAV immunogenicity, organoids, microphysiological system, organ-on-chips, body-on-a-chip

## Abstract

The FDA has predicted that at least 10-20 gene therapy products will be approved by 2025. The surge in the development of such therapies can be attributed to the advent of safe and effective gene delivery vectors such as adeno-associated virus (AAV). The enormous potential of AAV has been demonstrated by its use in over 100 clinical trials and the FDA’s approval of two AAV-based gene therapy products. Despite its demonstrated success in some clinical settings, AAV-based gene therapy is still plagued by issues related to host immunity, and recent studies have suggested that AAV vectors may actually integrate into the host cell genome, raising concerns over the potential for genotoxicity. To better understand these issues and develop means to overcome them, preclinical model systems that accurately recapitulate human physiology are needed. The objective of this review is to provide a brief overview of AAV gene therapy and its current hurdles, to discuss how 3D organoids, microphysiological systems, and body-on-a-chip platforms could serve as powerful models that could be adopted in the preclinical stage, and to provide some examples of the successful application of these models to answer critical questions regarding AAV biology and toxicity that could not have been answered using current animal models. Finally, technical considerations while adopting these models to study AAV gene therapy are also discussed.

## Introduction – gene therapy today

Over the past two decades, gene therapy has evolved from a hypothetical concept to a clinical reality for numerous genetic disorders, fulfilling its promise of a single-treatment cure in some cases (1). As of June 23, there were 7 gene therapy products approved by the FDA, several of which use CAR-T cells (2), and it is predicted that 10-20 products will be approved by 2025 (3).

Gene therapy involves introducing a functional copy of a gene into a cell to compensate for a defect in its own endogenous copy of that gene or to suppress a defective gene that encodes a harmful product. While gene therapy can take many forms to achieve its objective, these forms all fall into one of the following categories: gene addition, gene silencing, and gene editing/gene replacement, and they are comprised of two components - the vector and its encoded payload, which can include the therapeutic transgene, appropriate regulatory elements to confer transgene expression, and, in the case of gene-editing, the necessary machinery to mediate this process, e.g., CRISPR/Cas9, ZFNs, TALENS, prime/base editors. The process of gene delivery itself can be achieved by exposing the desired target cells to the vector *ex vivo*, and then infusing the cells to act as vehicles to carry the therapeutic gene, or by directly administering the vector *in vivo*. Success of gene therapy is primarily dependent upon the selection of an appropriate vector that can deliver its payload to the desired location with high efficiency (and ideally specificity, as well). For this to occur, the vector must also have the ability to evade barriers imposed by the host’s immune system.

Among the myriad gene delivery platforms available, vectors based upon viruses remain the most widely used. Millions of years of evolution has honed the innate capacity of viruses to transfer their genetic material into host cells, and this ability can be exploited to achieve delivery of therapeutic genes at far higher efficiency than with any nonviral vector that has been created to-date. Viral vectors used for gene therapy can be classified into integrating and non-integrating vectors. The choice is made depending on the desired expression profile (transient or prolonged), the size of the genetic payload (each virus has an ideal genome size which must be observed for efficient packaging), and the inherent biology of the target cell type/tissue (is the cell quiescent or dividing, is it short- or long-lived, which viral receptors does it express on its surface, etc.). Currently, the viral vectors that have shown the most promise in preclinical studies and in clinical trials are those based upon adeno-associated virus (AAV), lentiviruses (i.e., HIV), and murine retroviruses (e.g., MMLV, MSCV). While lentiviral and murine retroviral vectors are most often used in the context of *ex vivo* gene delivery, modifying cells *in vitro* for subsequent infusion, AAV has proven highly successful in an *in vivo* setting (4).

## AAV gene therapy

### Clinical landscape

Due to the tremendous potential it has demonstrated in both decades of preclinical studies and in past and ongoing clinical trials, AAV is currently a very popular choice for accomplishing *in vivo* gene delivery. Clinical data covering over 3000 patients treated over more than 20 years has demonstrated that AAV is a safe and well-tolerated gene delivery vector that can be highly effective (5, 6). Moreover, depending upon the target cell/tissue, AAV can achieve long-term transgene expression after a single infusion, with the longest reported period of > 15 years in primates (7). There are only two FDA-approved AAV gene therapy products on the market today (Luxturna and Zolgensma), but there are ~136 ongoing clinical trials involving AAV products to treat 55 different diseases (*ClinicalTrials.gov*). It is also expected that 50 trials will be completed in the next 3 years, of which 80% will be phase 2 (5). Currently, inherited disorders affecting the eye, lysosomal storage, and coagulation (hemophilia A and B) account for the majority of clinical trials. Among these, hemophilia A has the most ongoing trials, most of which are in late phases of development and a high likelihood of commercialization. Of the many ongoing clinical gene therapy trials, the vast majority involve gene addition - only 3 gene silencing and 3 gene editing trials are currently ongoing. For more detailed information on the clinical landscape of AAV, the reader is referred to the very thorough recent reviews by Au et al. and Kuzmin et al. (5, 6).

### AAV biology

AAV is a non-enveloped virus with an icosahedral capsid of approximately 22 nm in size. Wild type AAV contains a 4.7 kb single-stranded DNA genome flanked by inverted terminal repeats (ITRs). The genome consists of the *rep* and *cap* genes, which are required for replication/packaging of the genome and synthesis of viral capsid, respectively. The *cap* gene gives rise to three viral capsid proteins (VP1-3) and assembly-activating protein (AAP) which, as the name suggests, helps in the assembly of 60 VP subunits at a 1:1:10 ratio of VP1:VP2:VP3. Nine variable regions are present on each subunit, and they determine the tropism and intracellular trafficking of the vector, as well as the serotype (4). These regions of the capsid proteins also serve as the recognition domains for neutralizing antibodies (Nabs) (8, 9). AAV has very high seroprevalence (40-70% of human population harbor neutralizing antibodies, depending upon the AAV serotype) (10–12). This is one of the motivations behind the search for novel naturally occurring variants, and the quest to create new bioengineered variants, that could evade the pre-existing immunity present in the majority of the population and thereby allow the widespread use of AAV-based vectors. Bioengineering approaches are also being undertaken in an effort to increase AAV’s transduction efficiency and to enable transduction of specific cell types *in vivo*. Such targeted transduction would increase both safety and efficiency, since limiting transduction to only the desired target cell would avoid transduction of undesirable cells/tissues (e.g., germline) and would ensure the entire administered dose of vector was delivered to the target cells, thus maximizing clinical benefit. Such targeting would also have the added safety benefit of being able to achieve therapeutic levels of transduction of the target cells/tissues with a lower dose of vector.

### Engineering AAV

When constructing a recombinant AAV vector to be used for gene delivery, the *rep* and *cap* genes are removed and replaced with a cassette containing the therapeutic transgene and a promoter to drive its expression, while the ITRs are conserved, since they are essential for vector genome packaging (4). Importantly, removal of the *rep* gene renders AAV replication-defective, greatly increasing the safety of AAV. Selection of the appropriate capsid and promoter are the first steps to designing an effective AAV-based gene delivery vector. Since the capsid protein determines both the tropism, and thereby which cells can/will be transduced, and the antigenic profile of the vector (and its potential for being eliminated by pre-existing immunity or triggering an immune response) (9), bioengineering strategies have focused primarily on genetically modifying the capsid protein to achieve desired characteristics for the specific disease setting being targeted (4). To-date, hundreds of natural and bioengineered AAV variants have been reported, and tremendous progress has been made towards enhancing tropism for specific cells/tissues and for evading pre-existing anti-capsid immunity (13–18).

The promoter provides the opportunity to restrict expression of the therapeutic transgene to certain tissues, or even to specific cell types. Choosing a tissue-specific promoter avoids off-target expression of the transgene in undesirable cells, e.g., antigen-presenting cells (APCs) that could lead to an immune response, elimination of transduced cells, and loss of therapeutic effect. On the other hand, the use of a promoter that has been engineered to drive high-level constitutive expression, such as CAG, CBA, and CMV, or simply including the appropriate enhancer regions, can enable higher-than-normal expression of the transgene, allowing one to achieve a therapeutic effect from even a small number of transduced cells. Each choice has its own pros and cons. More than 25 trials have used tissue-specific promoters (5), yet these studies have shown that off-target effects still occur, due to “leakiness” of the promoter, allowing its promiscuous expression in non-target cells. Moreover, these studies have demonstrated the use of a tissue-specific promoter also results in a marked reduction in the effective dose of vector that reaches the target tissue, due to transduction of cells that will not contribute to expression of the transgene. While the ability to manufacture larger quantities of AAV has enabled the use of native promoters to drive more physiologically-relevant/appropriate expression of the therapeutic transgene, recent preclinical studies have suggested this approach can lead to severe toxicity due to the high vector copy number needed to achieve sufficient expression (19). As a result, the trend has been to rely on strong, constitutively active promoters, with 45% of trials between 2015 and 2019 employing the CAG, CBA, or CMV promoters discussed above (5). The use of one of these promoters does not guarantee success, however, as the high expression levels of such promoters often triggers their transcriptional silencing by methylation. In addition, supraphysiologic levels of expression of the transgene often come at the expense of reduced expression of endogenous genes that are forced to compete for limiting resources within the cells, leading to cell stress (6).

In addition to altering the capsid and the transgene cassette, the design of the vector backbone also provides an engineering opportunity to improve transduction efficiency. AAV naturally exists in a form with a signle-stranded DNA genome. After entering the target cells, the single-stranded AAV genome, upon reaching the nucleus, relies on the host cell’s machinery to be converted to a double-stranded form that can be transcribed. This conversion is a rate-limiting step in the transduction process. To attempt to overcome this bottleneck, researchers have developed what are known as “self-complementary” AAV vectors, the genome of which is double-stranded by design. As such, the vector genome undergoes transcription immediately upon nuclear entry, thereby increasing transduction efficiency and also decreasing the dose of vector required (20). This increase in transduction efficiency does not, however, come without a cost. By rendering the AAV genome self-complementary, the carrying capacity of resultant AAV vector is halved, severely limiting which transgens can be delivered by these vectors. In addition, some studies employing self-complementary AAV vectors have reported an increase in the innate immune response following transduction (21, 22). At the present time, the majority of the clinical trials are using AAV vectors with a single-stranded genome (6).

### Hurdles to be overcome with AAV gene delivery

The simplicity of its genome organization, which facilitates engineering and production, its ability to mediate sustained transgene expression in a variety of quiescent cells, its wide range of tissue tropism and high efficiency of transduction, and its established safety profile all contribute to the extensive application of AAV in gene therapy (23, 24). Despite its many advantageous properties, however, it is also important to acknowledge aspects of AAV that hinder its more widespread clinical use in gene therapy trials and pose potential risks to subjects receiving AAV vectors.

(i) Immunogenicity/seropositivity: Two of the major hurdles to the use of AAV-based vectors in clinical gene therapy are its high degree of immunogenicity and the fact that, depending upon the AAV serotype in question, 40-70% of people in the general population harbor neutralizing antibodies (NAbs), depending upon the AAV serotype (11, 12, 20, 25, 26). At the present time, clinical trials avoid the issue of pre-existing immunity to AAV by simply excluding any patients that harbor NAbs to the serotype of AAV being used as a vector. As a result, the vast majority of patients who could benefit from gene therapy are ineligible to receive this potentially life-saving treatment. Even in patients with no pre-existing immunity, a single injection of AAV can trigger a robust immune response to both the AAV capsid and the transgene product, causing cell-mediated elimination of transduced cells, loss of therapeutic effect, and potentially lead to toxicity in the transduced tissue (27–34). To prevent this deleterious cascade arising from T cell-mediated immunity, many AAV clinical trials pre-emptively administer a course of corticosteroids to the patients (32). However, novel strategies are constantly being developed (14, 16) to overcome these issues. These strategies run the gamut, starting at the design level with mutating critical antigenic sites on the capsid to generate novel AAV variants with no seroprevalence, all the way to strategies that are only incorporated at the time of AAV administration, such as chemical shielding of AAV antigens, elimination of antibody-producing cells, or removal of NAbs through plasmapheresis or using the IgG-degrading enzyme, IdeZ (35). While promising, these approaches are still in early stages of development and will require extensive preclinical testing and validation before clinical implementation can be considered.
(ii) Genomic integration: Wild-type AAV integrates into a specific locus (AAV-S1) on human chromosome 19, through actions of the *rep* gene (36–38). Unlike wild-type AAV, replication-defective AAV vectors lack the *rep* gene, and as such the majority of genomes that entered the target cell were assumed to remain episomal (39). However, studies over the past 2 decades have provided evidence that host (both animal and human) cell DNA-modifying enzymes can mediate genomic integration of AAV vectors, albeit at very low frequency (39–41). The extremely low incidence of genomic integration in these studies provided a sense of security with respect to the potential for AAV vectors to cause genotoxicity. The low likelihood of this risk being of concern for human patients receiving AAV vectors was further supported by a number of studies performed in juvenile and adult mice, all of which demonstrated a lack of AAV-induced mutagenesis in large cohorts of animals followed for relatively long times (23, 42–46). Unfortunately, however, the story proved to be very different when therapeutic, and even reporter-encoding, AAV vectors were administered to neonatal (1-2 day-old) mice, with some studies reporting AAV-induced hepatocellular carcinoma (HCC) in 100% of recipients (47–54). Interestingly, the majority of the AAV integrations that were present in the tumors were in the Rian (RNA imprinted and accumulated in nucleus) locus, which is orthologous to the human long non-coding RNA MEG8, increased expression of which has been correlated with poor prognosis in HCC patients (55). Needless to say, these findings raise significant concerns for human safety. Although to-date no AAV-induced genotoxic events have been confirmed in humans, these findings highlight the need for more in-depth studies in human-based systems to better determine the true risk of AAV-mediated genotoxicity in human patients, and the impact that the patient’s age may have on this risk. This need is further underscored by an elegant 10-year follow-up of AAV-mediated FVIII expression in adult dogs, which detected >1700 integration events and evidence of clonal expansion in liver tissue (56) and the recent demonstration that a powerful a new multiplex linear amplification-mediated polymerase chain reaction (M-LAM-PCR) assay was able to identify integration events and persistence of these integrated AAV genomes in both nonhuman primate tissues and human clinical liver biopsies post-AAV gene therapy (57, 58).
(iv) Tropism/Vector Biodistribution: Given these afore-detailed limitations and the lack of data in human-based model systems that accurately recapitulate normal physiology, it is not surprising that AAV clinical GT trials are experiencing immunological and/or inflammatory responses and levels of transgene expression that are often lower than what was expected based on preclinical studies (31, 59–64). Another issue that has hampered the translation of AAV vectors from animal models into human patients is the marked species-species differences that exist in AAV vector tropism (65), which precludes extrapolation of results between species and raises the critical question of how accurately even the best animal models can predict tropism/vector biodistribution, transduction efficiency, and eventual treatment success in humans. AAV’s tropism is governed by the specific molecular interaction between the viral capsid and moieties on the surface of the cell, with the interactions differing by serotype (66). Cellular uptake occurs after initial binding to moieties such as glycans and proteoglycans that are ubiquitously expressed in cells (67). This is followed by a series of secondary protein interactions which facilitate internalization (66–71). While species-species variation in tropism can be explained, in part, by differential expression of these receptors on animal vs. human cells, it has also been observed to be due to differences in capsid sequence which could vary the processing of the virus after its entry into the cell, thereby affecting eventual gene transfer (72). For example, studies have shown serotype-dependent difference in hepatic transduction efficiency may not always be due to the ability of different serotypes to enter hepatocytes but may instead be due to variation in post-entry processing such as translocation to the nucleus and conversion of single-stranded genome to double-stranded (59, 72–75), again emphasizing the importance of performing studies in the specific human tissues and cell types to be targeted to be able to draw meaningful conclusions and accurately predict clinical outcome. Since it is difficult to obtain multiple biopsies from patients in the clinical setting, especially those with hemophilia, which account for a substantial percentage of the ongoing trials, information on tropism and vector biodistribution has not been reported in detail in any published clinical trials to-date (29–32, 76), leaving this critical safety and efficacy issue unanswered.

In an effort to fill this void, Lisowski et al. (77) and others (78, 79) have made use of FRG^®^ mice whose liver was partially repopulated with human hepatocytes to evaluate the clinical utility of natural and genetically-engineered variants/serotypes of AAV These studies led to the identification of two variants that transduced human hepatocytes with high efficiency *in vivo*, AAV5 and AAV-LK03, both of which are currently being used in liver-directed clinical GT trial for hemophilia A. These data represent an important first step to defining the relative efficiency with which various AAV serotypes transduce human liver. However, because the only humanized tissue was the liver (specifically, only the hepatocytes) (77, 78), it is impossible to derive information on the specificity of these AAV serotypes for hepatocytes, *i.e.*, the true tropism in humans, only the efficiency with which they transduce these cells when there are no other human cells to compete for binding. As such, this model system leaves the critical questions of off-target effects and dilution of effective vector dose from uptake by non-target cells unanswered.

## Novel models to combat hurdles of AAV gene therapy

Although animals and humanized animals provide invaluable model systems for preclinical evaluation of gene therapy-based treatments, their failure to accurately recapitulate the myriad nuances of human responses to gene therapy has often led to unexpected outcomes, with respect to both efficacy and safety, during clinical trials. These shortcomings highlight the need to develop novel preclinical models that can more accurately recapitulate human physiology and genetics to replace, or at least supplement, existing models in preclinical studies, providing information of direct human relevance. A similar concern exists in the pharmaceutical world, where even the best animal surrogates often fail to accurately predict efficacy and toxicity of new drugs (80–83). To overcome the inherent problems with extrapolating animal data to humans, the WFIRM-led X.C.E.L. program created an integrated microfluidic human “body-on-a-chip” (*hBOAC*) platform, in which up to 12 different human 3D organoids retain normal physiology/functionality for weeks to months, that can be used to screen new drugs, define effects of environmental toxins/stressors, and test countermeasures (84–94). This novel system was able to predict toxicities of former FDA-approved drugs that were recalled due to unexpected human toxicities, sometimes only years after market release (84, 85, 94), demonstrating the immense potential of individual 3D human organoids and the integrated *hBOAC* platform to provide information that is otherwise not attainable with current animal models, and thus fill this critical knowledge gap and enable more direct translation of preclinical findings with new pharmaceuticals into clinical success. In the following sections, we will discuss how we envision this promising platform being adopted for use in gene therapy studies to enable more accurate predictions of the human response to gene therapy with respect to transduction efficiency, tissue and cellular tropism, and immunogenicity, as well as serving as a model in which to study the incidence and genomic location of AAV vector integration events in the context of an all-human system.

### 3D organoids

Organoids, while fairly new to the fields of biomedical and pharmaceutical research, originated several decades ago in the developmental sciences, born from the need for a model that could allow the study of the complex process of organogenesis (95). Organoids are three-dimensional (3D) structures derived from tissue-specific cells that self-organize and undergo spatially-restricted lineage commitment to recapitulate the structural characteristics, cytoarchitecture, and functional properties of the organ/tissue from which they are derived (96, 97). For example, human intestinal organoids have been shown to carry out key functions of the gastrointestinal tract including establishment and maintenance of an appropriate epithelial barrier, mucus production, absorption, and secretion of biomolecules (98). As detailed in the informative review by Hofer et al. (97), organoids have now been developed, representing all of the major human tissues. A critical scientific advance that greatly facilitated the fabrication of various organoids possible was the advent of induced pluripotent stem cells (iPSCs), which allowed generation of various highly-specialized cell types (99) which would otherwise be inaccessible. The availability of these tissue-specific cells allowed fabrication of more complex organoids that more closely replicate the anatomy and physiology of the native tissue. Moreover, iPSC technology has made possible the generation of patient-specific and disease-specific organoids, opening whole new avenues in research and drug testing. Aside from iPSCs, cells used to fabricate organoids include embryonic stem cells, cell lines, and adult stem/progenitor cells, and tissue-specific primary cells derived from tissue samples. To create organoids, cells are usually combined together in physiologically relevant percentages within a protein-rich medium that resembles the composition of the natural extracellular matrix (ECM) of the specific tissue to be modeled. The organoids can either be formed through self-aggregation to yield spheroids or by immobilizing the cells within a hydrogel matrix. The architectural complexity can be further increased by fabricating in a system that allows stratification, e.g., including a membrane to create an air-liquid interface in the case of lung organoids.

### Microphysiological systems (MPS) and organ-on-a-chip

MPS are microfluidic systems fabricated at a biologically relevant scale with the application of fluid flow and mechanical stressors to create a dynamic environment that more accurately recapitulates the physiology and microenvironment of the native tissue. The inclusion of these additional makes it possible to generate biomolecular gradients and to create mechanical cues such as shear stress and contraction/relaxation, allowing them to reproduce more of the physiological subtleties that are unique to each tissue than conventional static cultures. By combining MPS-based models and organoids, one obtains what are referred to as an “organ-on-a-chip”; integrating multiple distinct organ-on-a-chip devices together and enabling tissue-to-tissue communication *via* shared circulating media yields what has been dubbed a “body-on-a-chip”. These systems have proven to be very powerful preclinical models for testing pharmaceuticals, reproducing such complex integrated multi-organ physiologic responses as metabolism of a drug by the liver, with the resultant metabolites then trafficking to other organs and exerting toxicity (84, 93, 94, 100). Their small size/scale allows for better control of the microenvironment and also requires minimal resources. The immense promise of these organ-on-a-chip and body-on-a-chip platforms as preclinical tools is eloquently stated in the recently published report by Marx et al. (100), in which the authors detail a roadmap to regulatory acceptance of organ-on-a-chip models and highlight the potential for such systems to supplement and, perhaps, ultimately replace animal models for preclinical testing of drug efficacy and toxicity. Organ-on-a-chip systems are also being integrated with artificial intelligence platforms, further increasing the predictive power of these models and preclinical data derived from them. It is envisioned that the organ-on-a-chip platforms will reach the necessary qualification level within the next decade and will dramatically decrease the use of animal testing. Currently, the success rate for drugs in clinical development is a meager 15%, due in large part to the discrepancies seen between results in an animals and those observed when the drug moves to clinical trials. It is anticipated that the adoption of these all-human organ-on-a-chip models in the preclinical drug development pipeline will greatly improve this success rate, enabling safer and more effective drugs to enter the market at a lower cost to patients who need them. Although originally developed for drug screening, in the next sections, we will highlight how we envision the field of AAV gene therapy can also greatly benefit from implementing these organ-on-a-chip platforms.

## Technical considerations for adapting organ-on-a-chip and body-on-a-chip models to study AAV gene therapy

**Figure 1:**
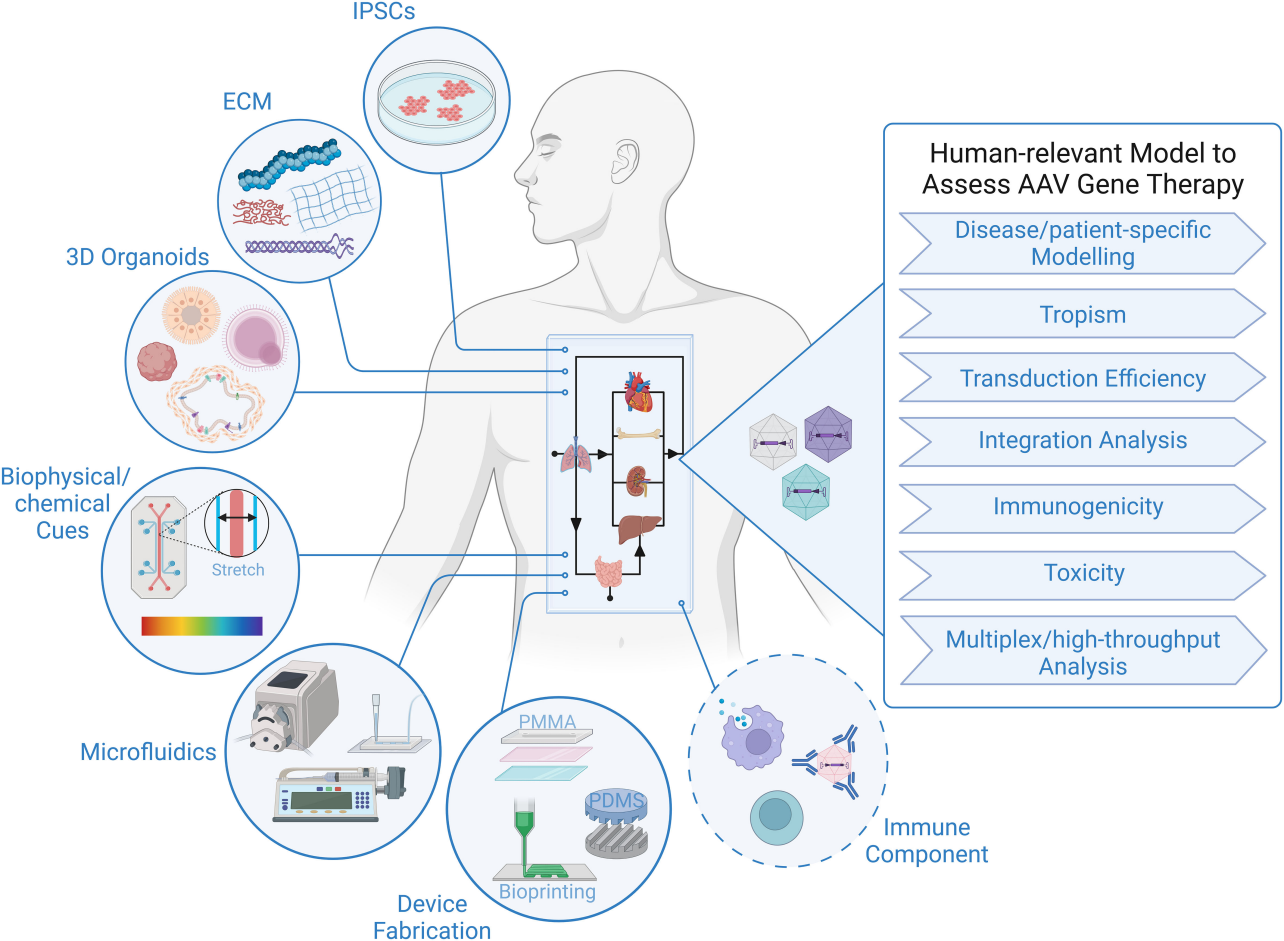
Human-relevant Preclinical Model to Accelerate AAV Gene Therapy Studies. Representation of critical components constituting body-on-a-chip platform that can be used to assess outcome of AAV gene therapy in the context of a human system. Addition of an immune component is essential to increase the predictive value of a model to study viral gene therapy.

The tremendous progress in biofabrication techniques has enabled the development of body-on-a-chip platforms and their use as preclinical models to probe human response to therapies to advance at an exponential rate in recent years (93, 101–104), and we foresee it only being a matter of time before these systems are extensively applied to gene therapy studies, because of their unique ability to provide answers to critical questions that elude even the best current models (**Figure 1**). Looking specifically at AAV-based gene therapy studies, it is important to identify technical considerations that are relevant to gene delivery. In general, a body-on-chip platform consists of 3 key components: (i) organoids formed with a normal repertoires of cell types in physiologically relevant frequencies; (ii) a universal media that is able to support all cell types present in each of the different organoids; and (iii) microfluidics and microfabrication techniques that enable physiological interaction of multiple organ compartments and create the necessary physical/chemical cues. In addition, it is often desirable to incorporate physical, biochemical, and optical sensing modalities to facilitate the performance of real-time, nondestructive analyses on the various organoids and their shared “circulation” (97). It is also important to consider possible interactions of AAV with any of the materials used to fabricate the device. For example, polydimethylsiloxane (PDMS) is a widely employed polymer with many attractive properties for microfluidic device fabrication, such as biocompatibility, low toxicity, optical transparency, elastomeric properties, gas permeability, ease of fabrication, and low manufacturing costs (105, 106). Unfortunately, it suffers from serious protein adsorption problems due to its hydrophobic nature (106, 107), a property that has the potential to result in binding of proteins of the AAV capsid and thereby interfere with transduction. Therefore, to adapt existing microfluidic “on-a-chip” platforms for studies employing AAV, it will likely be necessary to either opt for an entirely different material or to develop a suitable anti-fouling surface-treatment to avoid vector adsorption to the PDMS. Luckily, such treatments are currently an area of very active investigation (106, 108).

As a completely human system, the organ-on-a-chip and body-on-a-chip platforms have the potential to accurately predict transduction efficiency of specific human cell types, tropism/selectivity of transduction when simultaneously presented with multiple human tissues/cells, true cell-specificity of promoters within human tissues/organs, and the incidence and precise genomic location of AAV insertion events within the human genome. There are also certain inherent design aspects of these platforms that are beneficial for gene therapy studies. For example, microfluidic devices function at a very low volume, and therefore, high vector-to-cell ratios/multiplicity of infection (MOI) can easily be achieved without the need for large quantities of viral vector. The small size also allows for high-throughput screening of vectors by connecting an array of devices, which increases the power of analysis with minimal resources (109, 110). The compartmentalization/stratification allows exploration of the impact different routes of gene delivery, e.g., systemic administration vs. direct injection into a specific target tissue, has on the efficiency and specificity of vector delivery to the desired target organ.

Although many sources of cells can be employed to create the organoids in the body-on-a-chip, iPSCs have some advantages with respect to gene therapy, as their use not only allows fabrication of a body-on-chip in which all tissues and all cells possess an identical genetic makeup, but it also provides the opportunity for rapid high-throughput screening of a diverse population (111), and it allows the derivation of patient-specific cells that can be used for disease modelling and developing personalized approaches to gene therapy that are optimized for the specific patient to be treated (103, 112). Due to the inability to biopsy most tissues in human patients who receive AAV-based gene therapy, insertion event data is simply not available for human subjects, with the exception of a single study (57). Human body-on-a-chip platforms provide the unique opportunity to define patterns of AAV genome insertion and answer thew critical question of whether the frequency and pattern of genomic integration vary as a function of the recipient’s age and the tissue that is targeted, as has been observed in mice (58). The use of patient-derived cells to create the body-on-a-chip would also make it possible to assess the specific patient’s risk of insertional mutagenesis. No other system is currently available that can screen for patient-specific genotoxic events and provide invaluable data concerning the underlying molecular mechanisms governing any observed patient-to-patient differences.

Since pre-existing anti-capsid immunity and the development of an immune response to the vector and transgene post-gene delivery are a major hurdle limiting more widespread clinical use of AAV vectors, the inclusion of an immune component to the body-on-a-chip would tremendously increase the utility of this platform and markedly enhance its preclinical value. In order for an AAV vector to successfully achieve long-term transgene expression *in vivo*, key immune-related roadblocks must be overcome: (i) the presence of pre-existing neutralizing antibodies (NAbs) against the capsid, the presence of which can markedly reduce transduction; (ii) triggering of an adaptive immune response and NAbs by the capsid, which can prevent future re-administration; (iii) triggering of a cytotoxic T lymphocyte (CLT)-mediated response by the capsid, which can lead to the elimination of transduced cells and loss of therapeutic effect; and (iv) the transgene product can induce B cell- and T cell-mediated responses that can lead to generation of antibodies and CTLs against the transgene product (113). While it was initially supposed that innate immunity would be transient and inconsequential for AAV-mediated gene therapy, it is now appreciated that both the innate and adaptive arms of the immune system pose formidable barriers to successful AAV (113). Despite their critical role in the human response to gene therapy and the ultimate success or failure or such treatments, immune components have thus far not been addressed in any detail in organ-on-a-chip or body-on-a-chip systems. Being hematopoietic in nature, all immune cells are derived from hematopoietic stem/progenitor cells (HSPC) that reside within the bone marrow. We and others have created human bone marrow-on-a-chip systems to gain insight into the complex process of normal and malignant hematopoiesis in an all-human system (86, 114–119). Despite being very new, these platforms have already provided an unprecedented view into the differing interactions of healthy and malignant human HSPC with specific cells of the various bone marrow niches, often in real-time, and molecules involved in these processes. However, they have not yet been utilized to study immune ontogeny, likely due, at least in part, to the need for T cell precursors to traffic to and mature within the thymus to achieve full function, and B cell precursors to mature within germinal centers in secondary lymphoid organs such as the lymph nodes, ileal Peyer’s patches, and the spleen. As such, a platform containing bone marrow, thymus, and one of the secondary lymphoid tissues would be needed to study T cell and B cell development and function “on-a-chip” – an accomplishment that is likely going to take quite a bit of work to make a reality. As a first step towards this goal, Seet et al. described (120) a serum-free, artificial thymic organoid (ATO) system that supports the efficient and reproducible *in vitro* differentiation and positive selection of conventional human T cells from HSPC. Moreover, they showed that ATO-derived T cells exhibited mature naive phenotypes, a diverse T cell receptor (TCR) repertoire and TCR-dependent function, and that ATOs initiated with TCR-engineered HSPC produced T cells with antigen-specific cytotoxicity. While these ATOs provide a robust tool for studying human T cell differentiation, they have yet to be incorporated into an on-a-chip platform, precluding their integration with other hematopoietic tissues. A further major step was very recently made towards achieving at least part of a functioning human immune system on-a-chip by Ingber and colleagues (121), who showed that primary human blood B- and T-lymphocytes autonomously assemble into ectopic lymphoid follicles (LFs) when cultured in a 3D extracellular matrix gel, and that B cells in these germinal center-like LFs exhibit plasma cell differentiation upon activation. Most excitingly, the authors demonstrated that when these human LFs-on-a-chip were inoculated with commercial influenza vaccine, plasma cell formation and production of anti-hemagglutinin IgG was observed, and a repertoire of cytokines similar to vaccinated humans was secreted over clinically relevant timescales.

It is clear that ongoing advances in the organoid field will one day make a human immune system-on-a-chip a reality (122). In the meantime, however, it is important to note that most tissues possess an intrinsic immune surveillance component in the form of tissue-resident antigen presenting cells (APC) such as dendritic cells (DC) and macrophages. One of the first steps in detection of foreign entities by the innate immune system is the recognition of structural motifs called pathogen-associated molecular patterns (PAMPs), by pattern recognition receptors (PRRs) expressed on APC. Thus, while it is not yet possible to incorporate an entire functioning immune system, simply including the appropriate APC in a given organoid/tissue-on-a-chip dramatically improves the predictive power of these models, as activation of any of the innate immunity pathways can readily be detected through differential expression of genes involved in innate immunity. For example, the liver-on-a-chip platform we developed includes Kupffer cells at a physiologically appropriate frequency to enable investigation of immune and inflammatory responses within this system (94).

## Current application of 3D organoids and MPS platforms to study AAV gene therapy

While organoids and MPS have already been used extensively in studies focused on drug testing, disease modelling, and personalized medicine (reviewed in (103)), to-date only a fairly limited number of reports have employed organoids to study AAV gene therapy (123–145). Most of the human organoid-based AAV studies thus far have been performed on retinal organoids (123–131). The initial focus on organoids designed to model the human retina can likely be attributed to several factors. The first of these is the eye’s presumed immune-privileged status, which has allowed effective *in vivo* gene transfer to this organ and at least partial phenotypic correction of several different defects with very minimal to no immune reactivity in both preclinical and clinical studies (146–148), although more recent reports have questioned the true immune-privileged status of the intraocular space and the lack of an immune response following AAV delivery to the eye (149). A second factor driving the relatively large number of studies exploring AAV gene delivery to the retina with human organoids is the availability of well-established *in vitro* models, many of which are comprised of all the major cell types in the eye and have the ability to build functional synaptic connections and recapitulate photosensitivity (123–131). A small number of studies have also been performed with AAV vectors in human cerebral organoids to determine the optimal capsid for achieving efficient transduction and transgene expression within the human brain, to develop novel means of combatting latent HIV infection within CNS glial cells, and to test the ability of AAV-based treatment to provide phenotypic correction in the setting of genetic diseases that impact the CNS (132–136). Lung and gut/intestinal organoids have also been utilized to successfully identify AAV serotypes that yield optimal transduction in the respective tissue, to create and test chimeric AAV/Bocavirus vectors, to test AAV/RNAi-based treatments for SARS-CoV-2, and to test the ability of AAV vectors to correct the cystic fibrosis phenotype, comparing results obtained in human organoids to those in CF mice (137–140). Organoids of the human kidney have also been employed to test the ability of a novel synthetic AAV vector to achieve efficient gene transfer to the mesenchymal cells of this organ (145). We and others have begun using human liver organoids to define the basic biology, therapeutic efficacy, and potential toxicity of liver-directed AAV gene delivery (141–144) (more detail appears in a subsequent section below). Collectively, these studies have employed human organoids to answer various questions regarding AAV biology and behavior in a human system, and they have provided valuable information regarding the expression of various AAV receptors and the transduction efficiency of different AAV serotypes/engineered variants. They have also begun to define the transduction pattern in organoids comprised of heterogeneous cell populations (vector tropism) and the expression profile and localization of transgene product (thereby testing the true selectivity of various “cell-specific” promoters), and they have explored some of the mechanisms of cell attachment of AAV vectors to different cell types within these human tissues. These studies have also used organoids to validate animal study outcomes in an all-human setting and to test novel approaches to gene delivery/editing to target infectious agents such as HIV and COVID-19 (132, 139). By using iPSCs, either derived from patients or engineered *via* CRISPR/Cas9 gene editing, to fabricate these organoids, investigators have provided critical proof-of-concept for the value of using organoids to model a specific disease, *e.g.*, retinitis pigmentosa (127), *CRX*-Leber congenital amaurosis (131), and lysosomal β-galactosidase deficiency (135), and test the therapeutic effect of AAV-mediated gene correction, often in a patient-specific, *i.e.*, personalized medicine, setting.

Surprisingly, despite the rapid transition from static organoids to organ-on-a-chip and body-on-a-chip platforms in drug-based studies, organoid studies investigating AAV gene delivery have thus far been limited to individual 3D organoids in static conditions with the notable exception of the recent report by Achberger et al. (150), in which 3D human iPSC-derived retinal organoids were incorporated into a dynamic environment in a microfluidic device to create a retina-on-a-chip, fabricated with more than seven different essential retinal cells. The microfluidic device enabled not only the incorporation of a dynamic environment, but also a dual chamber setup, one housing the organoids and the other acting as vasculature. These two chambers were separated by a membrane barrier to protect the organoids from shear force. The compartmentalization and vasculature-like perfusion of the system enabled a physiological subretinal-like injection of the AAV particles and a nutrient supply *via* a choroidal-like vasculature. Seven variants of AAV were characterized for cell tropism and transduction efficiency, with the optical accessibility of this novel platform providing the opportunity for *in situ* live-cell imaging. This study represents the first time a human iPSC-derived organ-on-a-chip was utilized to test transduction efficacy of AAV gene therapy using a highly translational route of administration, and as the authors convincingly argue, the presesented data demonstrate the immense potential of human organ-on-a-chip (and ultimately body-on-a-chip) models as the next generation of screening platforms for future gene therapeutic studies.

Another key area in AAV biology and AAV-mediated gene transfer for which organoids and tissue-/body-on-a-chip platforms are ideally suited is the identification of existing AAV serotypes or the engineering of novel AAV capsid variants that enable passage of AAV across biological obstacles such as the blood-brain-barrier (BBB) and the pulmonary epithelial barrier (PEB). As many diseases could benefit from efficient delivery of AAV vectors to the brain or the lung, the identification of AAV serotypes/variants that can efficiently cross the BBB or PEB would have a substantial clinical impact. Human brain organoids that accurately model the functionality of the BBB have already been developed and used extensively to study the ability of therapeutics to access the central nervous system (89, 151–155), to better understand alterations to the BBB that occur in various disease settings and during infection and the neuroinflammation that ensues due to BBB disruption (89, 153, 156–162), to study tumor metastasis (163–168), to examine receptor-mediated antibody transcytosis (169–171), and to develop and test novel nanoparticles for their ability to transit the BBB (172, 173). Similarly, lung organoids have also been used to study epithelial barrier function under normal homeostasis and in response to infection, exposure to nanomaterials, environmental toxicants, and small particulates, as well as for screening of new pharmaceuticals (174–185). Despite the fairly widespread use of organoids and tissue-/body-on-a-chip systems to study barrier function and its impact on health, disease, and pharmacological intervention, the use of these systems to explore AAV’s ability to transit these key biological barriers represents a highly promising, but is as-yet unexplored, avenue of research.

To our knowledge, we are the only group to-date to use organoids to begin to study the human immune response to AAV vectors. These studies, which are still in the early stages, utilized human liver organoids comprised of all major cell types present in the human liver in physiologically relevant frequencies: 78% human primary hepatocytes, 2% biliary cells, 5% hepatic stellate cells, and 5% endothelial cells, and included 10% Kupffer cells, enabling the evaluation of innate immune responses. Importantly, these liver organoids recapitulate the function of the native liver – hepatocytes express p450 reductase and secrete serum albumin, are tightly interwoven with stellate cells expressing vimentin, and exhibit microvilli, and bile canaliculus-like structures form within 7 days, demonstrating a rudimentary biliary system. Moreover, these organoids metabolize drugs, respond to inflammatory stimuli by initiating fibrosis and steatosis, and they demonstrate acetaminophen-induced toxicity that is reversed by its clinical countermeasure N-acetyl cysteine (84, 92–94, 186, 187). Based on the accuracy with which it recapitulates *in vivo* biology and function, we hypothesized that these liver organoids could provide a unique and powerful system in which to define the cellular, molecular, and functional impacts of transduction of the human liver with AAV3b and AAV5 to model ongoing clinical gene therapy trials using these serotypes of AAV to treat hemophilia A (188, 189), and to determine the true tropism of these serotypes in this all-human system. Our results to-date have shown the utility of this human liver organoid platform to determine the cellular tropism, transduction efficiency, and potential of these two clinically employed serotypes of AAV to induce inflammation and innate immune response following liver-directed gene transfer (142). Studies are currently ongoing to transition to a body-on-a-chip platform with a simulated circulatory system to better replicate the systemic *in vivo* AAV gene delivery being employed in hemophilia A gene therapy clinical trials and to incorporate the bone marrow-on-a-chip we have developed (4) and either the lymphoid follicle (LF)-on-a-chip described by Goyal et al. (121) or the tonsil organoids Wagar et al. (190) used to characterize the varying response donors with differing immune status (vaccination status, age, immunosuppressive state, *etc.*) exhibited to influenza, which we predict will greatly enhance the amount and quality of the data concerning the immune response to AAV that can be garnered from this platform.

## Discussion and future directions to further improve preclinical AAV studies

The objective of this review has been to provide an overview of AAV gene therapy, to underscore its immense therapeutic potential, and to discuss the hurdles that currently limit its more widespread application to the correction of genetic diseases in a larger percentage of patients who would benefit from such intervention (**Table 1**). We highlighted how the lack of robust preclinical models that can accurately recapitulate human biology and thus predict clinical response is one of the main limitations that needs to be addressed. To progress from this impasse, we discussed how 3D organoids, MPS, and organ/body-on-a-chip platforms can sere as powerful alternatives to current preclinical models and how they have already begun to do so in the realm of pharmaceuticals. To support this tenet, we provided examples of successful application of these techniques to answer questions that animal models have failed to adequately and accurately address. In addition to the advantages stemming from their being an “all-human” model, we also stressed the ability of these on-a-chip systems to enable the creation and exploration of disease-specific and/or patient-specific models for personalized medicine and the further value such options brings to these models. We also provided a brief list of technical considerations to be applied in the context of assessing gene therapy using organ/body-on-a-chip platforms, including the need to incorporate a functional immune component, which would exponentially increase the value of these models. Despite the many unique advantages that organoids and tissue-/body-on-a-chip platforms possess compared to traditional 2D culture systems and animal models, it is important to acknowledge that they are not without limitations. The first of these, as we have discussed, pertains to the need to integrate a more physiologically relevant and functional immune component to better model the *in vivo* setting and accurately predict the potential for an immune response and/or inflammatory reaction following delivery of a given AAV vector. Second, while the use of microfluidic allows the organoids/tissue chips to communicate with one another and approximates a very primitive circulatory system, to truly model the human body, it will be necessary to create media that accurately models the components of human serum and to devise a means of coating the microfluidic channels with appropriate endothelial cells to recreate the important biological effects the reticuloendothelial system exerts on vectors and other agents that are administered systemically. The third issue that bears mention is the source of cells used to create the organoids/tissue-on-a-chip. While iPS cells are certainly convenient and enable the investigator to generate essentially any cell type that is needed, one must very carefully interpret results obtained with iPS-derived cells, as they often possess a phenotype that is developmentally immature and may not accurately model an adult patient (191–194). In addition, the source of cell used to generate the iPS cells can also exert an effect on the phenotype and function of the subsequently differentiated tissue-specific cells as a result of the irreversible epigenetic “memory” each cell type possesses (195–198). Even when the appropriate cell types are used to create the organoid/tissue-on-a-chip, the most complex physiological aspects and cell-cell communication networks of certain tissues (the brain for example) will likely be almost impossible to recapitulate with complete accuracy *in vitro*. As these technologies continue to mature, however, it is safe to assume that the fidelity with which these systems mirror their *in vivo* counterparts will steadily improve. With these caveats in mind, we hope this review has served to highlight the immense potential organ-on-a-chip and body-on-a-chip platforms have to revolutionize preclinical testing, and that the examples we have provided have convinced the reader that these systems represent a powerful translational model that can provide critical information regarding transduction efficiency, tropism, potential immunogenicity, and genotoxicity, thus greatly aid in translating safer and more effective AAV-based gene therapies to the clinic to treat/cure a wide array of genetic disorders.

**Table 1:** Strategies of studies employing organoids and microphysiological systems to overcome the hurdles of AAV gene therapy.

Hurdles of AAV gene therapy	Novel models to overcome AAV gene therapy hurdles (Organoids and microphysiological systems)	References
**Potential Immunogenicity**	• Gene expression profiling to assess activation of innate immunity pathway	(142)
**High Seroprevalence**	• Screening/identification of novel variants using organoid platform	(125, 128–130, 132, 134, 137, 138, 144, 150)
**Accurate Prediction of Transduction Efficiency/Tropism**	• Physiologically relevant transduction efficiency prediction with models that are entirely human compared to “humanized” animal models • Cell-type-specific immunostaining to define tissue/cell tropism	(123–130, 132–140, 142–144, 150)
**Genomic Integration**	• High-throughput integration site analysis to determine frequency/loci of inserts and clonal dominance	(142)
**Toxicity**	• Viability • Tissue-specific toxicity assessment (Liver – ALT/AST, fibrosis, etc.)	(128, 133, 142)

## Author contributions

RR wrote original draft of manuscript. CP, AA, and GA-P edited and added to the draft to yield the final document. All authors contributed to the article and approved the submitted version.

## Funding

The authors declare that this study received funding from Pfizer Global Medical Grants System; grant #61589649. The funder was not involved in the study design, collection, analysis, interpretation of data, the writing of this article, or the decision to submit it for publication.

## Conflict of interest

The authors declare that the research was conducted in the absence of any commercial or financial relationships that could be construed as a potential conflict of interest.

## Publisher’s note

All claims expressed in this article are solely those of the authors and do not necessarily represent those of their affiliated organizations, or those of the publisher, the editors and the reviewers. Any product that may be evaluated in this article, or claim that may be made by its manufacturer, is not guaranteed or endorsed by the publisher.
